# Spinodal Decomposition in Fe-25Cr-12Co Alloys under the Influence of High Magnetic Field and the Effect of Grain Boundary

**DOI:** 10.3390/nano8080578

**Published:** 2018-07-28

**Authors:** Lin Zhang, Zhaolong Xiang, Xiaodi Li, Engang Wang

**Affiliations:** Key Laboratory of Electromagnetic Processing of Materials (Ministry of Education), Northeastern University, No. 3-11, Wenhua Road, Shenyang 110819, China; xzl5612@163.com (Z.X.); lixiaodi310@163.com (X.L.)

**Keywords:** nanosized particle, grain boundaries, high magnetic fields, precipitation

## Abstract

Fe-Cr-Co alloys precipitate nanosized α_1_ particles through spinodal decomposition, and their magnetic performance is susceptible to influence by the shape and arrangement of α_1_ particles. We studied spinodal decomposition during the heat treatment of Fe-Cr-Co alloys by both experimental and numerical simulation. Fe-Cr-Co alloys were fabricated first by directional solidification, followed by thermomagnetic treatment in a high magnetic field (HMF) and step aging. The experimental results show a spinodally decomposed structure consisting of nanosized α_1_ particles. The applied HMF caused the α_1_ phase to change into a rod-like shape. Moreover, a feather-like structure was observed near the grain boundary (GB), with slim α_1_ rods regularly arranged along the direction perpendicular to the GB. With the shape change and alignment of the α_1_ phase in the HMF, Fe-Cr-Co alloys show magnetic coercivity that is superior to those of samples without an HMF. To reveal the influence of HMF on phase transformations and the effect of GB, we conducted phase-field simulations of spinodal decomposition in the Fe-Cr-Co alloy. A migrating GB contributes to the elongation and arrangement of the α_1_ phase in the regions where the GB has passed. Thus, the α_1_ phase is arranged as parallel rods that are perpendicular to the GB. This GB effect consists of the effect of enhanced atomic mobility and the elastic energy. The α_1_ rods are elongated along the direction of HMF. The simulation results indicate that the feather-like structure is caused by a combined effect of both the GB and HMF. It is shown that the model generates microstructures which are qualitatively similar to those observed experimentally.

## 1. Introduction

Fe-Cr-Co alloys are a group of semi-hard permanent magnetic materials. The significant advantage of these alloys is the low-cost shaping possibilities. However, Fe-Cr-Co alloys have a critical problem: their performance is susceptible to influence by the shape and arrangement of nanosized α_1_ particles, which are precipitated due to spinodal decomposition, and the kinetics of this process are not fully addressed because of the complex influencing factors, such as the external magnetic field and lattice defects. 

An ideal microstructure contains ultrafine elongated particles arranged regularly with a preferential direction. Thus, a magnetic field is applied in the heat treatment of an Fe-Cr-Co alloy to acquire anisotropic structures with elongated α_1_ particles regularly arranged in an α_2_ matrix [1]. The two phases each have a different saturation magnetization. The anisotropy resulting from heat treatment in the magnetic field is proportional to the square of the difference between the magnetizations of the two phases. The magnetic field exerts its effects through magnetization to accelerate the precipitation of α_1_ phase. Thus, the enhancement of magnetic field strength is a possible means to further improve the anisotropic structure in an Fe-Cr-Co alloy. Recent experimental studies have included the use of an HMF to form a regular structure in Fe-Cr-Co [2]. The magnetic properties increase with increasing external magnetic field intensity in the range below 8 kOe. However, the magnetic properties decrease with increasing intensity in the range higher than 10 kOe [3]. 

In addition to the magnetic field, lattice defects, such as dislocations and grain boundaries, play an important role in spinodal decomposition. Moore et al. demonstrated experimentally the effect of precipitate plates on spinodal decomposition in an Al-Ag alloy [4]. Besides, experimental studies have reported that microstructures resulting from spinodal decomposition change near the boundary in some alloys, such as Fe_2_NiAl [5], Au-Ni [6], Cu-Ni-Fe [7], and Cu-Ni-Sn [8] alloys. Published evidence has demonstrated the boundary effect on spinodal structures. However, the complexity of this process has not been fully addressed, and the spatially distributed characteristics cannot be captured through purely analytical approaches.

In recent years, different numerical approaches have been developed for the prediction of spinodal decomposition with boundary effects. Solute-dislocation interactions have been studied by phase-field modeling approaches with varying degrees of sophistication [9,10,11]. Moreover, Ramanarayan and Abinandanan [12,13,14] studied the effect of enhanced atomic mobility on spinodal decomposition at the GB, and revealed that the GB becomes a transformation front, whose migration leaves behind a structure consisting of alternating lamellae of A-rich and B-rich phases. Also, Li et al. [15] investigated the effect of dislocations and GBs on spinodal decomposition in an Fe-Cr alloy, and demonstrated that a dislocation stress field facilitates spinodal decomposition. 

In the heat treatment process of Fe-Cr-Co alloys, magnetic fields, grain boundaries, and stress-strain fields all contribute to the total energy. The physical processes underlying these effects are not yet fully understood, especially the elastic effect of dislocation in a migrating boundary. We annealed Fe-Cr-Co alloys in a high magnetic field and found a feather-like microstructure near the GBs. To discuss and understand the formation mechanism of the feather-like structure, we simulated the spinodal decomposition under the influence of a migrating GB by the phase-field method, considering the effect of HMF, the elastic energy, and the enhanced mobility at the GB region. 

## 2. Materials and Methods 

**Figure 1:**
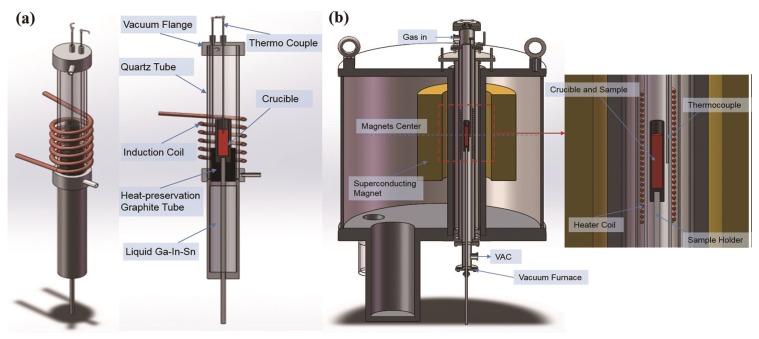
Schematic diagram of the experimental apparatus: (**a**) Directional solidification; (**b**) Heat treatment in a high magnetic field.

Fe-25%Cr-12%Co (mass) alloys were prepared by melting pure iron, chromium, and cobalt metals in an electric-arc furnace under a high-purity argon atmosphere. Then, the ingots were cut and enveloped in an alumina crucible with an inner diameter of 8 mm and a length of 100 mm for the directional solidification experiment. The samples were melted by conduction coils and directionally solidified by pulling the crucible assembly downward at a constant speed (400 μm/s) by means of synchronous motor into a water-cooled cylinder containing liquid Ga-In-Sn metal (Figure 1a).

**Figure 2:**
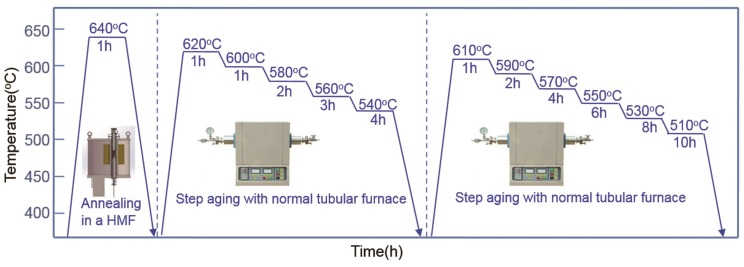
Schematic illustration of heat treatment used for the experiment.

The cast specimens were given an initial solution treatment at 1300 °C for 1 h and subsequently quenched in water. Then, the specimens were given a thermomagnetic treatment at 640 °C for 1 h in a vacuum furnace in a 12 T high-magnetic field (Figure 1b). After this, Fe-Cr-Co alloys were subjected to step aging twice, first in the range of 540–620 °C, and then in the range of 510–610 °C. Figure 2 presents the heat treatment routes. This heat treatment route was designed by reference to the work of some researchers [16,17], and amended due to a series of preliminary experiments. 

Transmission electron microscopy (TEM) was carried out by a FEI Tecnai G^2^ 20 transmission electron microscope (FEI Technologies Inc., Hillsboro, OR, USA). Magnetic properties were measured in a Lakeshore 7407 Vibrating Sample Magneto meter (Lake Shore Cryotronics Inc., Westerville, OH, USA) in fields up to 2 T, and the magnetic field during measurement was applied parallel to the direction of the annealing HMF. 

## 3. Experimental Results

**Figure 3:**
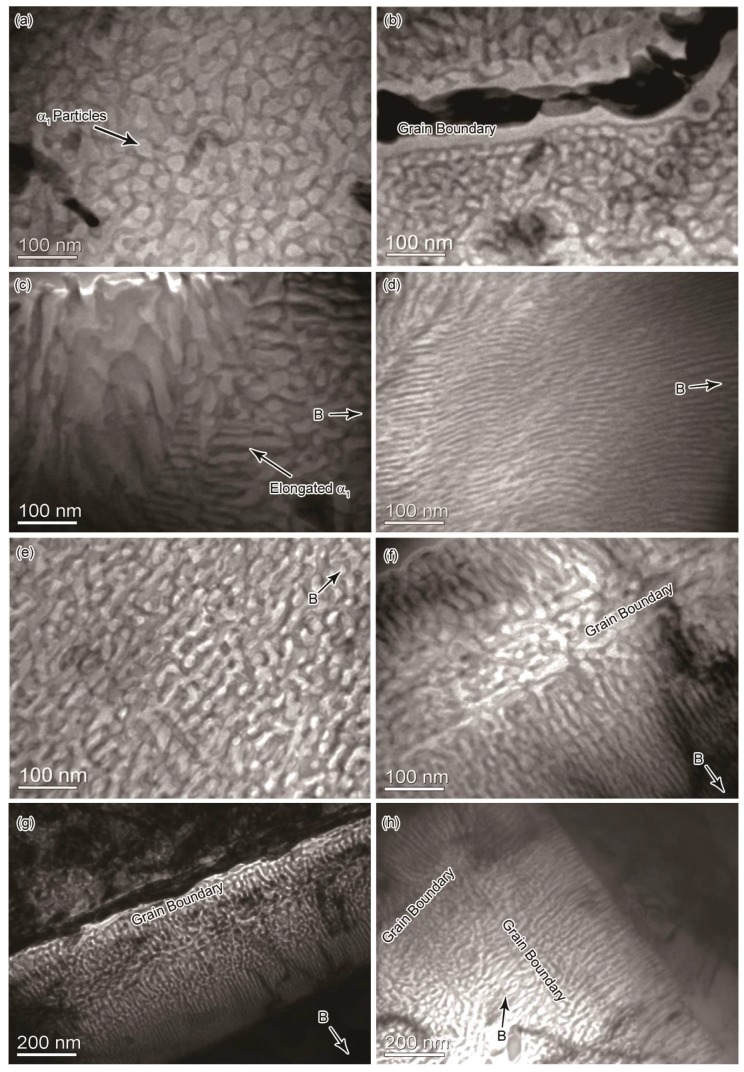
TEM image of the Fe-Cr-Co alloy after thermomagnetic treatment and step aging: Annealed without magnetic field, inside a grain (**a**) and near a GB (**b**), respectively; annealed in a 1 T magnetic field, rod-like α_1_ particles (**c**) and feather-like structure (**d**), respectively; annealed in a 12 T magnetic field, inside a grain (**e**) and near a GB (**f**), respectively. (**g**) and (**h**) show a long GB and a GB at the grain corner in the 12 T sample, respectively.

We examined the microstructure of the Fe-Cr-Co alloys that were processed with annealing and two-step aging. We found a common spinodally decomposed structure with dispersive nanosized α_1_ precipitates throughout the material, as evident in the TEM image in Figure 3. The shapes of α_1_ particles appear as irregular dots in the samples annealed without the magnetic field (Figure 3a). Besides, the grain size ranges from 10 μm to 100 μm. The GBs appear to influence the particle size in the surrounding regions. A clear GB is shown in Figure 3b, and the particles close to the GB have a smaller size compared with the ones far from the GB. 

When an external magnetic field was applied during the annealing stage, the form of the α_1_ phase changed into rod-shaped precipitates, and they were aligned in a preferential direction. The rod-like α_1_ particles are shown in Figure 3c,e, which shows the grain interior far from the GB. On the other hand, a kind of feather-like structure is observed to distribute preferentially near the GBs in samples annealed in the HMF, as shown in Figure 3d in the case of the 1 T HMF, and as shown in Figure 3f in the case of the 12 T HMF. The feather-like structure contains preferentially aligned α_1_ long rods (Figure 3d). The α_1_ rods are aligned perpendicular to the GB (Figure 3f), which means the formation of the feather-like structure has a directional relation with the GB. Figure 3g shows a long GB at lower magnification, and a region of the feather-like structure is shown to have α_1_ long rods aligned perpendicular to the GB. Figure 3h shows a GB at the grain corner, illustrating α_1_ rods growing perpendicular to the GB on both sides of the corner.

We tested the composition of the α_1_ and α_2_ phases using Energy Dispersive X-ray Spectroscopy (FEI Technologies Inc., Hillsboro, OR, USA) at the position shown in Figure 3e. The composition of the α_1_ phase is 63.13% Fe and 12.89% Co (mass), and the composition of the α_2_ phase is 56.89% Fe and 9.75% Co. The α_1_ phase is FeCo-rich and thus has a much higher magnetization than the α_2_ phase. When a magnetic field is applied during the heat treatment, this magnetization difference leads to the preferential growth of α_1_ precipitations in the direction of the magnetic field.

**Figure 4:**
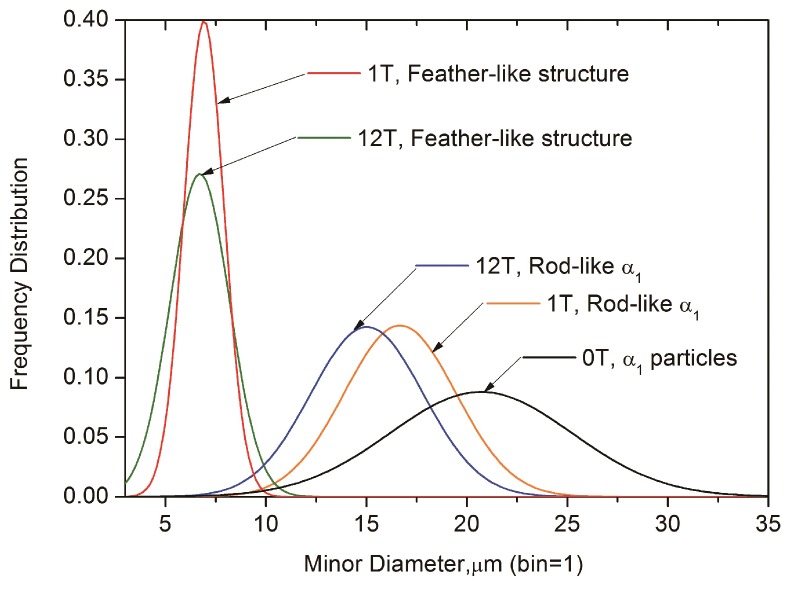
The frequency distribution of diameter for α_1_ rods in Fe-Cr-Co alloys, the normal distribution curves were fitted according to the measured data.

Figure 4 shows the distribution of the diameter for the α_1_ rods in different experimental conditions. The fitted curves are based on the measured diameter data in the experimental microstructure, and all these curves appear to be Gaussian distributions. In the regions far from the GB, the particles have a maximum frequency of around 21 nm diameter (mean of the distribution) in the sample without the HMF, and the curve tails off at around 35 nm. On the other hand, the rod-like particles have a mean diameter of around 17 nm in the presence of an HMF of up to 1 T, and around 15 nm in the presence of an HMF of up to 12 T. Also, the distribution range of the diameter is decreased by applying the HMF. Obviously, the HMF leads to a decrease in diameter of the α_1_ rods, having a stronger effect with increased magnetic flux density. In the regions with the feather-like structure, the long rods have a mean diameter of around 7 nm in both the case of 1 T HMF and 12 T HMF. This mean diameter decreases considerably compared with that in regions far from the GB. Moreover, the distribution of the α_1_ rods in feather-like structures have a much smaller range and a higher maximum frequency compared with that in other regions.

**Figure 5:**
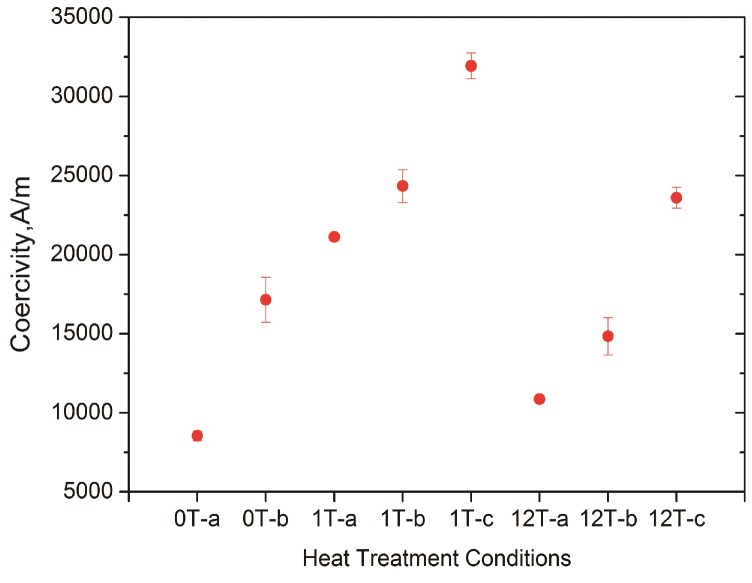
Coercivity of Fe-Cr-Co alloys subjected to different thermomagnetic treatments: Horizontal ordinate shows the conditions of thermomagnetic treatments, where 0 T, 1 T, and 12 T denote the magnetic flux density in the annealing process; a and b denote one- and two-step aging without HMF, respectively; c denotes step aging twice in an HMF, with the same magnetic flux density that was applied during its annealing process.

We investigated the magnetic properties of the Fe-Cr-Co alloys and found that the magnetic coercivity changes considerably with the conditions of the thermomagnetic treatments, as shown in Figure 5. Because of the alignment of the α_1_ rod-like particles in the HMF, Fe-Cr-Co alloys show magnetic coercivity that is superior to those of samples without HMF. The coercivity is 8551 A/m in the sample subjected to an annealing treatment followed by step aging (0T-a). The coercivity increased to 21110 A/m when the annealing treatment was carried out in a 1 T magnetic field (1T-a), implying that the HMF promotes the enhancement of coercivity during the annealing process. However, the coercivity tends not to change proportionally with the magnetic flux density, as the coercivity decreased to 10864 A/m in the case of a 12 T magnetic field (12T-a). This change is consistent with the results achieved by Sun et al. [3], in which the authors attributed the decline of magnetic properties to the decrease of shape anisotropy of the α_1_ phase. A magnetic field of an excessively high intensity is supposed to restrain the coarsening process and form tiny α_1_ particles with smaller aspect ratios. 

The coercivity increased further when processing step aging for a second time, as can be seen in Figure 5 (0T-b, 1T-b, and 12T-b). Moreover, a further increase in the coercivity was achieved by applying an HMF during step aging (1T-c and 12T-c). The contribution of the feather-like structure to the magnetic properties might be small, since it only occurs near the GBs and occupies a small portion of the structure. This is because the solidification and annealing conditions in this experiment led to large grains. The experimental results indicate a route to fabricate a feather-like structure that encompasses a larger portion, but this requires a new fabrication method that can generate smaller grains with more GBs. The refinement of the grains could possibly cause the GB to have a strong effect on the anisotropic structure and the magnetic properties.

The experimental results here show that the size and shape of the α_1_ rods were influenced by the magnetic field and defects such as the GB. In the next section, we discuss the formation process of the elongated α_1_ rods under different influencing factors.

## 4. Phase-field Simulation of Spinodal Decomposition

We observed a new feather-like structure in the experiment and found that the formation of this structure is related to the GB and the HMF. To analyze this effect, we present the results of phase-field simulations of the spinodal decomposition in the Fe-Cr-Co alloy during thermal-magnetic treatment and compare with experimental results.

### 4.1. Theoretical Model

We employed the phase-field method and use the nonlinearized Cahn–Hilliard diffusion equation to describe the time evolution of a composition field in the Fe-Cr-Co system. The model for the spinodal decomposition process used in this study is a combination of several models, including the models of Cahn and Hilliard [18,19], Ramanarayan and Abinandanan [12], Biner [20], and Lv and coworkers [21]. Our model uses a composition field c, and the time-dependent Cahn–Hilliard equation is written by [19]:

(1) $$\frac{\partial c}{\partial t}=\nabla \cdot \left(M\nabla \frac{\delta E}{\delta c}\right)\;$$

where *E* represents the total free energy, and *M* is the atomic mobility tensor, which may depend on position. The mobility value at the boundary is considered to be much higher than that of the grain interior. The enhanced mobility is calculated here by an equation simplified from the model of Ramanarayan and Abinandanan [12]. A position-dependent mobility tensor M in a direction parallel to the GB plane is defined by:

(2) $$M=M_{b}+\varphi_{g}M_{t}\;$$

in which the value of factor $\varphi_{g}$ is 1 at the GB and is zero outside the GB. *M_b_* is the atomic mobility in the grain interior. *M_t_* is the enhanced atomic mobility in the plane of the GB. 

Thermomagnetic treatment is important in the spinodal decomposition process of the Fe-Cr-Co alloy. We considered the effect of a magnetic field on the phase separation behavior, in addition to the chemical potential energy. Also, we considered the role of elastic inhomogeneities on the phase separation behavior. The total free energy *E* is assumed to be additive and composed of three parts in our model: chemical, elastic, and magnetic energies. The Cahn–Hilliard equation has been modified as:

(3) $$\frac{\partial c}{\partial t}=\nabla \cdot M\nabla \left(\frac{\delta E^{ch}}{\delta c}+\frac{\delta E^{el}}{\delta c}+\frac{\delta E^{mag}}{\delta c}\right)\;$$

where *E^ch^*, *E^el^*, *E^mag^* are the chemical energy, the elastic energy, and the magnetic energy, respectively. 

The chemical energy *E^ch^* is expressed in a simple Cahn–Hilliard equation [19]:

(4) $$E^{ch}=\int_{V}\left[\frac{\delta f}{\delta c}+\frac{1}{2}\kappa {\left|\nabla c\right|}^{2}\right]dv\;$$

where *κ* is the gradient energy coefficient. In this paper, a double-well function is used to describe the bulk free energy *f(c)*:

(5) $$f_{}\left(c\right)=A_{c}c^{2}{\left(1-c\right)}^{2}\;$$

in which *A_c_* is a positive constant. 

We simulated the boundary elastic stress on the phase decomposition by incorporating into our calculations the model of Biner [20], which considers the stress fields of dislocations that compose the boundary. For simplicity, the boundary is supposed to be composed of an array of dislocation dipoles (composed of two edge dislocations). The dislocation dipoles were introduced on *a* (1 −1 0) slip plane with Burgers vector *b* = *a*_0_/2 [1 1 1], where *a*_0_ is the lattice constant. The elastic energy can be described by [20]:

(6) $$E^{el}=\frac{1}{2}\int_{V}\sigma_{ij}\varepsilon_{ij}^{el}dv\;$$

(7) $$\varepsilon_{ij}^{el}=\varepsilon_{ij}-\varepsilon_{ij}^{0}\;$$

where $\sigma_{ij}$ is the stress, $\varepsilon_{ij}$ is total strain, and $\varepsilon_{ij}^{0}$ is the position- and composition-dependent eigenstrain. The total strain $\varepsilon_{ij}$ is related to the displacements *u_i_* and is given by the kinematic equation, expressed as:

(8) $$\varepsilon_{ij}=\frac{1}{2}\left[\frac{\partial u_{i}}{\partial x_{j}}+\frac{\partial u_{j}}{\partial x_{i}}\right]\;$$

where *u* and *x* are the displacement and position vectors, respectively. All the phases in Fe-Cr-Co alloys are assumed to be linear elastic. The stresses $\sigma_{ij}$ are obtained through Hooks law $\sigma_{ij}^{}=C_{ijkl}\varepsilon_{ij}^{el}$, where C_ijkl_ is the position-dependent elastic modulus tensor. In this paper, the elastic coefficients for bcc Co are assumed to be $C_{ij}^{Co}=C_{ij}^{Fe}$, and the elastic coefficients for Fe and Cr are $C_{11}^{Fe}=228.0$ GPa, $C_{12}^{Fe}=132.0$ GPa, $C_{44}^{Fe}=116.5$ GPa, $C_{11}^{Cr}=350.0$ GPa, $C_{12}^{Cr}=67.8$ GPa, and $C_{44}^{Cr}=100.8$ GPa, respectively [22].

The system requires the solution of mechanical equilibrium,

(9) $$\frac{\partial \sigma_{ij}^{}}{\partial x_{i}}=0\;$$

The contribution of dislocations to the elastic energy is calculated through their eigenstrain [20]:

(10) $$\varepsilon_{ij}^{0}=b⊗n=(b_{i}n_{j}+b_{j}n_{i})/2d\;$$

where *b* is Burgers vector to the slip plane, *n* is the normal vector to the slip plane, and *d* is the interplanar distance of the slip planes.

*E^mag^* is the magnetic energy, which depends on the magnetic domain morphology in the microstructure and the external magnetic field. The magnetic energy contributing to total free energy is taken as [21]:

(11) $$\frac{\delta E^{mag}}{\delta c}=\mu_{mag}=\mu_{ext}^{mag}+\mu_{exch}^{mag}+\mu_{an}^{mag}+\mu_{d}^{mag}\;$$

in which $\mu_{ext}^{mag},\mu_{exch}^{mag},\mu_{an}^{mag}$, and $\mu_{d}^{mag}$ are the chemical potentials corresponding to the external magnetic field energy, magnetic exchange energy, magnetocrystalline anisotropy energy, and demagnetizing energy, respectively.

In this model, we simplified the calculation of magnetic energy from the model of Lv and coworkers [21]. The magnetocrystalline anisotropy and the magnetic exchange energy was neglected here. The demagnetizing energy is the dipole–dipole interaction energy between magnetic moments, which is expressed as [21]:

(12) $$E_{d}=-\frac{1}{2}\int_{V}I_{s}{\vec{H}}_{d}\cdot \vec{m}(\vec{r})dv\;$$

where *I_s_* is the absolute value of the magnetization moment vector, which depends on the local composition. The vector *m(r)* is the normalized magnetization moment (|*m(r)*|=1). ${\vec{H}}_{d}$ is the demagnetizing field, which is calculated by [21]:

(13) $${\vec{H}}_{d}({\vec{r}}_{i})=-\frac{1}{4\pi \mu_{0}}\nabla_{\vec{r}}\int_{V}\frac{1}{\left|\vec{r}-{\vec{r}}_{i}\right|}\nabla_{\vec{r}}\cdot \left(-{\vec{I}}_{s}(\vec{r})\right)dv\;$$

The external magnetic field energy is expressed as:

(14) $$E_{ext}=-\int_{V}I_{s}\vec{H}\cdot \vec{m}(\vec{r})dv\;$$

were $\vec{H}$ is the external magnetic field.

We introduce the following dimensionless parameters: $M^{*}=M/M_{b}$, where *M_b_* is the atomic mobility in the grain interior, $M_{b}=1$; $t^{*}=t\cdot M_{b}f_{0}/b^{2}$, in which *f_0_* is a reference energy, $f^{*}=f/f_{0}$; the dimensionless gradient energy coefficient $\kappa_{c}^{*}=\kappa_{c}^{}/(f_{0}b^{2})$, $b=L/L^{*}$, b is the magnitude of Burgers vector *b* = *a*_0_/2 [1 1 1]. In this study, only the external magnetic field energy and demagnetizing energy was considered, and the magnetic chemical potential is calculated by [21]:

(15) $$\frac{\delta E_{mag}^{*}}{\delta c}=K_{mag}\mu_{}^{mag,*}=K_{mag}(-{\vec{H}}_{ext}^{*}({\vec{r}}^{*})\cdot m({\vec{r}}^{*})\frac{\partial I_{S}^{*}}{\partial c}-K_{d}{\vec{H}}_{d}^{*}({\vec{r}}^{*})\cdot m({\vec{r}}^{*})\frac{\partial I_{S}^{*}}{\partial c})\;$$

(16) $${\vec{H}}_{d}^{*}({\vec{r}}_{i})=-\nabla_{}^{*}\int_{V}\frac{1}{\left|{\vec{r}}^{*}-{\vec{r}}_{i}{}^{*}\right|}\nabla_{}^{*}\cdot \left(-{\vec{I}}_{s}^{*}({\vec{r}}^{*})\right)dv^{*}\;$$

(17) $$K_{mag}=2A^{\prime }I_{s,\max}^{2}/(f_{0}b^{2})\;$$

where *I_s,max_* is the maximal value of *I_s_*, $I_{s}^{*}=I_{s}/I_{s,\max}^{}$, $H_{ext}^{*}=H_{ext}/(2A^{\prime }a^{2}I_{s,\max})$. $K_{d}=1/8\pi \mu_{0}A^{\prime }a^{2}$, where A is the magnetic exchange constant 

The dimensionless grid sizes were set to be $\Delta x^{*}=1.0$ and $\Delta y^{*}=1.0$ in the simulations. The dimensionless time step $\Delta t^{*}$ was set as 10^-3^. The simulation cell had *N_x_*= *N_y_*= 800 grid points with grid spacing d*x* = d*y* = 1. The width of the dipole (width of boundary) was 20 grid-spacing. The concentration field c was initially set to have random initial fluctuations of about *c*_0_ = 0.4, and periodic boundary conditions were used in the simulation, which were set the same as in [2]. Since this study is a combination of several models, the parameters related to the magnetic field contribution were taken from [2], the parameters related to the enhanced mobility were taken from [12], and the parameters related to the dislocations in the GB were taken from [20]. We wrote the simulation codes by Matlab.

### 4.2. Calculated results and Discussion

As discussed above, there are several different factors acting on the spinodal decomposition of Fe-Cr-Co alloys. To understand the mechanism of this process, we designed several conditions to observe the microstructural evolution through the phase-field simulation. (1) The first condition is the single effect of the enhanced mobility (EM); (2) The second condition is the combined effect of EM and elastic energy of dislocation (EED), by which we tried to compare the effect of EED to EM; (3) The third condition is to observe the effect of an applied HMF, combined with the same parameters set for the second condition. The dislocation dipoles in the second and the third conditions have an equal spacing. Such an arrangement of dislocation exists in a tilt GB; (4) The fourth condition has the same parameters as the third condition, except the spacing between dislocation dipoles is unequal. Since the dislocation spacings in a real GB cannot always be equal, we changed the dislocation spacing to be randomly distributed in a range, to observe the dependence of the EED effect on the dislocation spacings; (5) In the fifth condition, we changed the direction of the HMF to orient parallel to the GB. 

**Figure 6:**
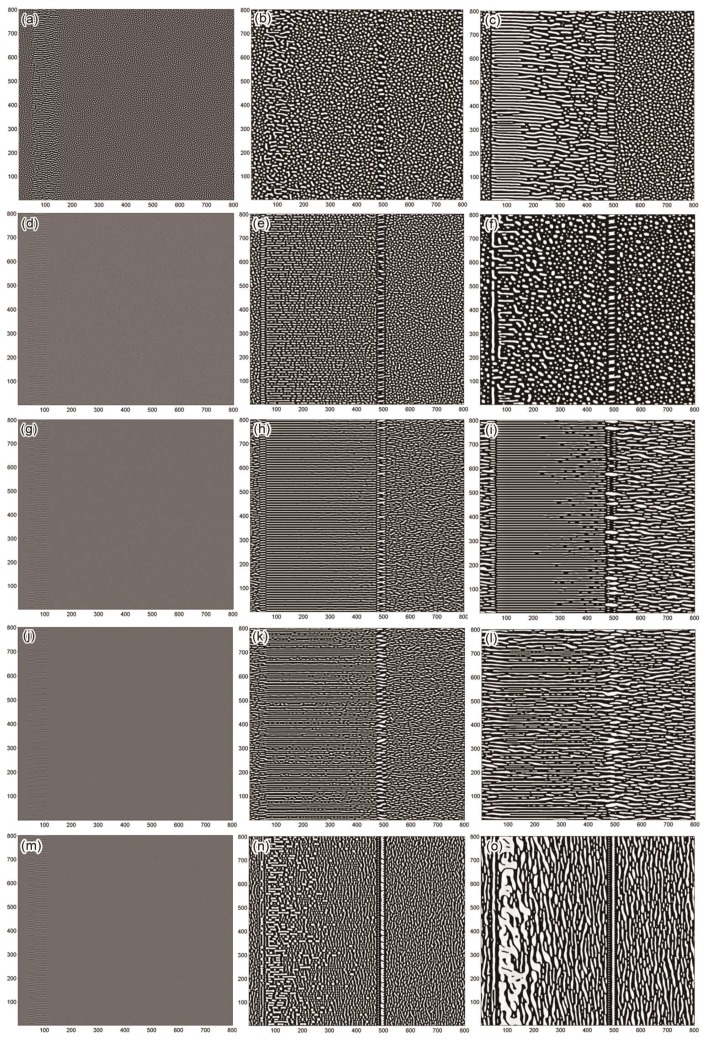
Time sequence of snapshots for the spinodal decomposition under different conditions. (**a**–**c**) First condition (EM): (**a**) *M_t_* = 5, t* = 2000; (**b**) *M_t_* = 5, t*= 20,000; (**c**) *M_t_* = 100, t*= 20,000; (**d**–**f**) second condition (EM + EED, *M_t_* = 5); (**g**–**i**) third condition (EM + EED + HMF); (**j**–**l**) fourth condition (EM + EED + HMF) with unequal dislocation spacing (range from 5 to 20 grid); (**m**–**o**) fifth condition (EM + EED + HMF), HMF parallel to the GB. In conditions 2–5, the images from left to right correspond to t* = 100; 10,000; and 50,000, respectively. This simulation used an 800 × 800 grid, with periodic boundary conditions along both *x* and *y* axes.

Figure 6 shows the two-dimensional simulation of the spinodal decomposition in the Fe-Cr-Co alloys under several different conditions in the thermomagnetic treatment. The white parts are the FeCo-rich regions (α_1_ phase), and the dark parts are the Cr-rich regions (α_2_ phase). The initial state of the supersaturated solid solution was set with a small composition fluctuation due to the computer-generated random number. Our simulation introduced an array of edge dislocation dipoles (20 grid-spacing in width and 10 grid-spacing apart in the y-direction) to represent a GB. The GB was set to migrate in the right direction (the left edge of GB migrates from *x* = 40 to *x* = 480, with a velocity of 5). 

In the first condition, the mobility in the GB region is enhanced in the direction parallel to the GB according to equation (2). Figure 6a–c shows the influence of enhanced mobility (EM) on the spinodally decomposed structure. In the region where the GB has passed, the α_1_ phase tends to change from round particles to rods. This trend is significant at the initial stage of the spinodal decomposition, as shown in Figure 6a when *M_t_*= 5, t* = 2000. There is competitive growth between the α_1_ rods accelerated by the GB and the α_1_ particles precipitated from other regions. As the α_1_ round particles from other regions grow to a certain size, the effect of EM cannot change the shape of these particles, as shown in Figure 6b. Moreover, the α_1_ rods might change into irregular shapes and lose the regular arrangement in the subsequent coarsening process. The effect of EM is more significant with high *M_t_*. Figure 6c shows the morphology under *M_t_*= 100, where α_1_ rods tend to be elongated and regularly arranged perpendicular to the GB. The diameter of the α_1_ rods appears to be approximately equal or larger compared with the α_1_ round particles without the EM effect. 

In the second condition, we set a normal effect of EM (*M*_t_ = 5) combined with the EED generated by dislocation dipoles with equal spacing of 10 grid. Figure 6d shows an initial state at t* = 100 of the supersaturated solid solution, and a trace of the GB can be observed on the left side of the image. The effect of EM and EED has accelerated the spinodal decomposition, leading to the prior precipitation of the α_1_ phase in the GB regions, making the trace of the GB clear. In the regions where the GB has passed, the α_1_ particles were elongated by the EED, arranged in lines that are perpendicular to the GB, as shown in Figure 6e. The combined effect of the EED and EM on the elongation of the α_1_ phase is more obvious compared with that of only EM in the first condition. The effect of the GB in the second condition is also limited by the competitive growth from the α_1_ particles formed by composition fluctuation before the presence of GB, and the elongation of the α_1_ phase only affected the initial stage of spinodal decomposition. These original rods tend to change into irregular shapes or round particles after being aged for a longer time, hence, the morphology in this region lost its regular arrangement, as shown in Figure 6f.

Figure 6g–i shows the effect of an external magnetic field being applied to the spinodal decomposition. Figure 6g shows a trace of the GB; as discussed above, the trace is formed by the prior precipitation of the α_1_ phase. Despite this prior precipitation being generated by the effect of EM and EED, a clearer trace of the GB is formed with an applied HMF, as shown in Figure 6g. Since the rod-like α_1_ precipitates in the trace are parallel to the HMF, their growth is improved by the magnetization. Figure 6h shows the morphology of two regions separated by the GB with differently shaped α_1_ phases. In the region where the GB has not passed, the α_1_ precipitates are elongated along the external magnetic field, and some appear as curved rods. Moreover, a feather-like structure is formed in the region where the GB has passed under the combined effect of the HMF, the EM, and the EED. The feather-like structure contains very long α_1_ rods that are regularly arranged, which is quite similar to the morphology observed experimentally in Figure 3d. In the coarsening process, the width of the α_1_ phase increases with increasing aging time. Nevertheless, the feather-like structure has a high structural stability and exhibits a slow coarsening rate. Therefore, after a long coarsening process, the α_1_ phase in the feather-like structure has a much smaller width compared with that in other regions, as shown in Figure 3i. The morphological developments of the simulated microstructures are in good agreement with the experimental results that were observed. 

We used the fourth condition to study the dependence of the EED effect on the dislocation spacings. Figure 6j–l shows the effect of a GB in which the dislocations are arranged with unequal spacings between them. The GB trace shown in Figure 6j exhibits this spacing difference. The feather-like structure is also formed in this condition, except that some parts of it appear to be discontinuous, which makes it not as regular as the one shown in Figure 6h. These discontinuous regions correspond to the traces of the large spacing between them. A spacing larger than 15 grid leads to a discontinuous distribution of the α_1_ phase and disturbs the formation of the feather-like structure, indicating there is a critical spacing required to form the feather-like structure. In the later coarsening process, these discontinuous regions disturb the stability of the feather-like structure, leading to a faster coarsening rate, as shown in Figure 6f. The large spacing in the fourth condition provides an extreme case; this spacing difference might be larger than that in a real boundary in the Fe-Cr-Co alloys. The experimental feather-like structure shown in Figure 3d,f both exhibit a more continuous and regular morphology than the simulated results of the fourth condition, though they are not as regular as the one simulated with the third condition. This indicates that the dislocation spacing difference is small in the GBs causing the experimental feather-like structure. 

In the fifth condition, we changed the direction of the HMF to orient parallel to the GB, and the other parameter settings are the same as in the third condition. At the initial stage, the effect of the GB is stronger, hence, Figure 6m appears to be similar to Figure 6g. Later, the HMF takes effect, whereas the elongation effect of the GB and HMF on the α_1_ phase cannot be combined. The formation of the feather-like structure has failed. Instead, there is competitive growth between the α_1_ precipitates accelerated by the GB and the α_1_ precipitates accelerated by the HMF, as shown in Figure 6n. They interfere with each other, with the α_1_ precipitates growing in a tilt direction in some regions. In the end, the effect of the HMF is advantageous, because it allows for the control of the growth direction of the α_1_ precipitates in most regions, as shown in Figure 6o. In addition, this mutual interference between the two effects results in a fast coarsening of some α_1_ precipitates with large size near the original position of the GB. A similar morphology is observed experimentally in the left part of Figure 3c.

**Figure 7:**
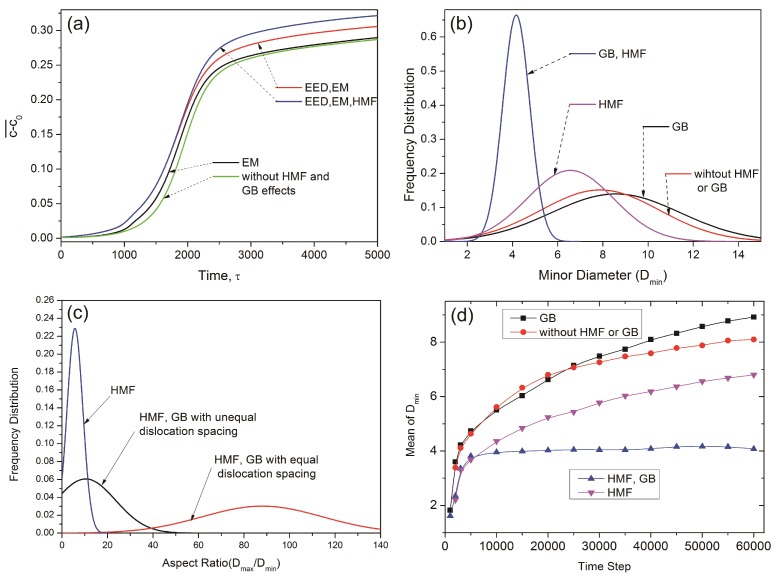
Plots of (**a**) change of average concentration $\bar{c-c_{0}}$ against time; (**b**) Frequency distribution of minimum diameter (*D_min_*); (**c**) Frequency distribution of aspect ratio (*D_max_ / D_min_*); (**d**) mean minimum diameter (*D_min_*) against time.

In this model, the total energy is influenced by several factors, including the HMF, the EM, and the EED. The decomposition rate appears to be different depending on the different combinations of these factors. The decomposition rate is indicated by the change of average concentration $\bar{c-c_{0}}$. Figure 7a shows the $\bar{c-c_{0}}$ curves against time, considering different factors. The $\bar{c-c_{0}}$ curve has the smallest value in the case excluding all the effects of the GB and HMF, corresponding to the slowest decomposition rate. The decomposition rate is enhanced slightly by considering the EM effect, and enhanced much further by considering the EED effect. This demonstrates that the dislocations accelerate the local precipitates of the α_1_ phases. The highest value of $\bar{c-c_{0}}$ is shown in the curve considering the effect of the HMF, indicating that the HMF can accelerate the precipitation of the α_1_ phase. 

The shape of the α_1_ phase includes spheres, ellipses, and rods, hence, we measured their size with both maximum diameter and minimum diameter. Figure 7b shows the distribution of the minimum diameter (*D_min_*) that corresponds to the different regions shown in Figure 6f,i. The largest value of the mean *D_min_* is shown in the curve considering only GB. Besides, this curve also has the largest distribution range. The *D_min_* decreased slightly after removing the GB factor, indicating that the GB promotes the coarsening of the α_1_ phase, probably through the enhanced mobility. Under the effect of the HMF, the mean *D_min_* and the distribution range are decreased considerably. The smallest value of the mean *D_min_* is shown in the curve representing a region with the combined effect of the HMF and GB, namely, the feather-like structure shown in Figure 6i. 

Figure 7c shows the aspect ratio of the α_1_ precipitates generated in different conditions, including the single effect of the HMF (namely, the non-GB regions in the third and fourth conditions), and the GB effect under an HMF with equal and unequal dislocation spacing (namely, the feather-like structure in the third and fourth conditions). Considering only the effect of the HMF, the aspect ratio ranges up to 20. Under the combined effect of the HMF and GB, the feather-like structure shows a considerable increase in the aspect ratio of the α_1_ rods. In the third condition, the aspect ratio ranges up to 140 in the feather-like structure. Although the GB has a weaker elongation effect in the fourth condition, the distribution of the aspect ratio still ranges up to 50.

The determination of the mean *D_min_* can be used to evaluate the coarsening rate of the α_1_ phase. Figure 7d shows the mean *D_min_* of the α_1_ precipitates against time in several conditions. The mean *D_min_* increases quickly at the initial stage of decomposition, then the coarsening rate slows down with increasing aging time. Considering only the GB, the precipitates have the highest coarsening rate, and the mean *D_min_* increases quickly. The coarsening rate decreases slightly after removing the GB factor. Considering the effect of the HMF, the coarsening rate becomes much slower. Furthermore, in the region considering the combined effect of the HMF and GB, namely, the feather-like structure, *D_min_* nearly stops increasing after time step 5000, indicating a high structural stability in this region. The high structural stability occurring in the simulation can explain the formation of the feather-like structure in the experimental results shown in Figure 3, in which the α_1_ phase has a much slimmer shape compared with other regions.

## 5. Conclusions

This study focuses on the spinodal decomposition of Fe-Cr-Co alloys during aging treatment under the effect of an HMF. The α_1_ precipitation changes from round particles to rod-like shapes under the HMF, with decreased minimum diameter. A feather-like structure is observed near the GB in samples with the HMF, containing long and slim α_1_ phase with a high aspect ratio. The formation mechanism of this structure was analyzed by phase-field simulation. The simulation generated microstructures which are qualitatively similar to those observed experimentally. The enhanced mobility and elastic energy generated by a migrating GB both promote the elongation and arrangement of the α_1_ phase. The simulation indicated an essential route to fabricate the feather-like structure by refinement of the grains. It is concluded that the feather-like structure occurs when the HMF is perpendicular to a migrating GB, and it depends on the combined effect of enhanced mobility, elastic energy in the GB, and the magnetic energy. The formation of the feather-like structure requires that the dislocation spacing is in an appropriate range. The feather-like structure has a high structural stability during the coarsening process, facilitating the preservation of the α_1_ phase’s slim form.
